# Radiation Damage in Biomolecules and Cells

**DOI:** 10.3390/ijms21218188

**Published:** 2020-11-01

**Authors:** Mario P. Carante, Francesca Ballarini

**Affiliations:** 1 INFN (Italian National Institute for Nuclear Physics), Sezione di Pavia, via Bassi 6, I-27100 Pavia, Italy; mario.carante@pv.infn.it; 2 Physics Department, University of Pavia, via Bassi 6, I-27100 Pavia, Italy

Ionizing radiation is widely used in medicine, both as a diagnostic tool and as a therapeutic agent. Furthermore, several exposure scenarios, like occupational and environmental exposure (e.g., radon), raise radiation protection issues, since the initial damage to DNA and chromosomes may lead either to cell death [1,2], which in turn can cause early tissue damage, or to cell conversion to malignancy and, eventually, cancer [3]. It is therefore mandatory for the scientific community to continuously update and improve the knowledge about the mechanisms governing the induction of radiation effects in biological targets, as well as to apply the acquired information to optimize the use of ionizing radiation and the corresponding radiation protection strategies.

Although the DNA double helix is widely recognized as the main radiation target, the features of the critical DNA damage type(s) leading to cell death or cell conversion to malignancy are still unclear. Moreover, the role played by other potential targets, which may be involved in bystander effects and other phenomena that may occur at low doses, deserves further investigation. Among the many possible medical applications, several aspects of cancer hadron therapy should be further addressed, including a more and more accurate RBE (Relative Biological Effectiveness) evaluation [4,5]. Such investigations can be carried out both experimentally, by means of in vitro and/or in vivo studies, and theoretically, by biophysical models and simulation codes.

In this framework, this Special Issue reports studies on the effects of ionizing radiation at molecular and cellular level, as well as possible applications in medicine and radiation protection. More specifically, some works [6,7,8] analyzed the early stages of the interaction between ionizing radiation and DNA, with particular attention to the role played by radiation track-structure in modulating the induction of DNA cluster damage, which is thought to lead to important endpoints including chromosome aberrations and cell death [9,10,11]. Another work [12] studied the impact of such damage on nuclear architecture, which is known to play a relevant role in DNA damage induction and repair [13]; other studies [14,15] investigated the consequences of hypoxia on the evolution of the initial damage, and one work analyzed genome-wide DNA alterations in X-irradiated human fibroblasts [16].

Among the studies aimed at better understanding the mechanisms involved in cancer radiation therapy, as well as improving its effectiveness, the metabolic response of glioblastoma multiforme (GBM) cells to photons and C-ions was characterized [17], the therapeutic potential of a new targeted c-SRC inhibitor molecule on X- or proton-irradiated GBM cells was investigated [18,19], the sensitization of breast cancer cells by checkpoint kinase 1 inhibition was studied [20], and the in vivo molecular response to proton therapy and its efficacy in a xenograft model was analyzed [21]. Furthermore, potential differences in the effects induced by passive or active clinical proton beams were explored [22], and FLASH radiotherapy was reviewed [23]. Within the field of cancer therapy side effects, the consequences of high radiation doses on gene expression in healthy tissues were analyzed [24] and the RBE for late effects in the central nervous system following proton or C-ion therapy was predicted by a novel biophysical model called BIANCA [25]. Since ionizing radiation can also be used to treat diseases other than cancer, radiation-stimulated translocation of CD166 and CRYAB proteins to the endothelial surface was found to provide potential vascular targets on irradiated brain Arteriovenous Malformations (bAVMs) [26].

Finally, in the framework of radiation protection studies, the protective role of melatonin and Vitamin D [27], as well as hypothermia [28], was reviewed, and valine radiolysis by fast ions was investigated [29]. Furthermore, the changes in radio-sensitivity and responses to ionizing radiation of embryos during the early developmental stages of the preimplantation were analyzed [30], and epigenetic modifications induced by ionizing radiation were critically reviewed [31].
